# Supplementary material for: Subcellular Localizations of Catalase and Exogenously Added Fatty Acid in Chlamydomonas reinhardtii
Source: Cells. 2021 Jul 30;10(8):1940. doi: 10.3390/cells10081940 (PMC8391285; doi:10.3390/cells10081940)
Supplement: Supplementary file 1 [file cells-10-01940-s001.zip › cells-1309984-supplementary.pdf]

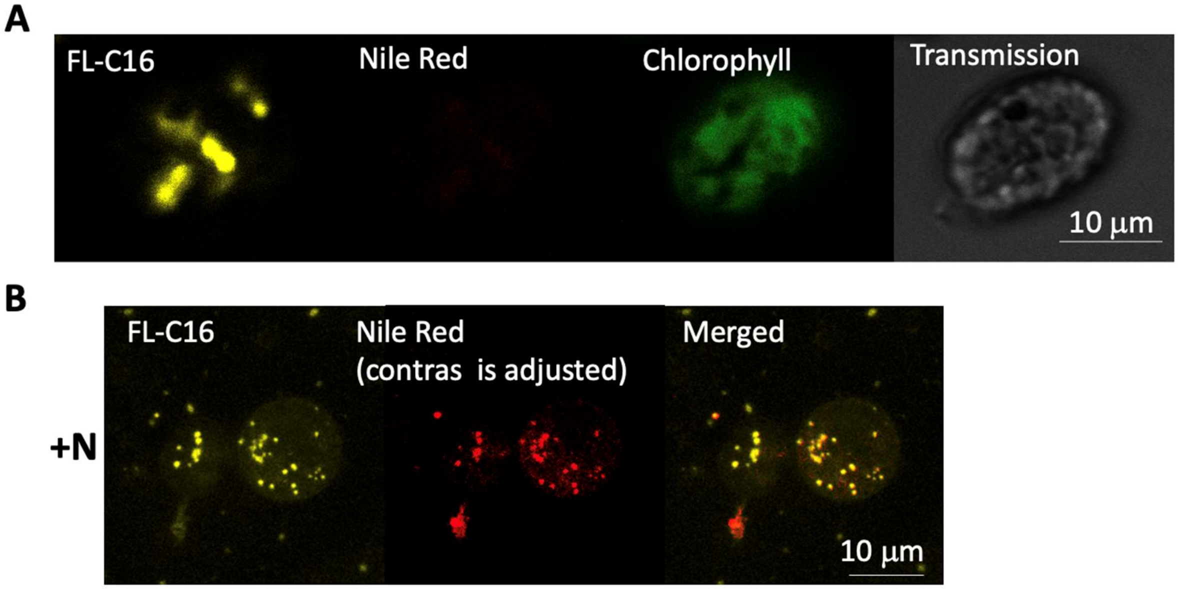

Figure S1. Little blead-through of signals between FL-C16 and Nile Red A. Control experiment for FL-C16 signals blead-through to Nile Red channel *C. reinhardtii* CC124 was cultured in TAP-N. FL-C16 (20 μM) was added to the culture medium 3 d before microscopy. Optical conditions identical with Figure 5 (Materials and Methods Table 2) were used to detect signals of FL-C16, Nile Red, and Chlorophyll without Nile Red staining. Note little blead through of FL-C16 signals to the Nile red channel. B. Co-localization of FL-C16 signals and reduced Nile Red signals in Figure 5 To clarify colocalizations of FL-C16 signals and that of reduced Nile Red signals in nitrogen repleted cells in Figure 5, brightness and contrast of the Nile Red signals were increased. Note signals of FL-C16 and those Nile red are colocalized.

Movie S1. 3-D view of *C. reinhardtii* cell expressing CIS2-CFP with FAIMs. Thirty optical sections of a 20 μm z-stack in Figure 4 were converted in 3-D view. Independent fluorescence channels of CIS2-CFP and FL-C16 are show.
